# dTULP, the *Drosophila melanogaster* Homolog of Tubby, Regulates Transient Receptor Potential Channel Localization in Cilia

**DOI:** 10.1371/journal.pgen.1003814

**Published:** 2013-09-19

**Authors:** Jina Park, Jeongmi Lee, Jaewon Shim, Woongsu Han, Jinu Lee, Yong Chul Bae, Yun Doo Chung, Chul Hoon Kim, Seok Jun Moon

**Affiliations:** 1 Department of Oral Biology, Yonsei University College of Dentistry, Seodaemun-gu, Seoul, Korea; 2 Department of Life Science, University of Seoul, Seoul, Korea; 3 Department of Pharmacology, Brain Korea 21 Project for Medical Science, Brain Research Institute, Yonsei University College of Medicine, Seoul, Korea; 4 Yonsei Institute of Pharmaceutical Sciences, College of Pharmacy, Yonsei University, Inchon, Korea; 5 Department of Oral Anatomy and Neurobiology, BK21, School of Dentistry, Kyungpook National University, Daegu, Korea; 6 Severance Biomedical Science Institute, Yonsei University College of Medicine, Seoul, Korea; New York University, United States of America

## Abstract

Mechanically gated ion channels convert sound into an electrical signal for the sense of hearing. In *Drosophila melanogaster*, several transient receptor potential (TRP) channels have been implicated to be involved in this process. TRPN (NompC) and TRPV (Inactive) channels are localized in the distal and proximal ciliary zones of auditory receptor neurons, respectively. This segregated ciliary localization suggests distinct roles in auditory transduction. However, the regulation of this localization is not fully understood. Here we show that the *Drosophila* Tubby homolog, King tubby (hereafter called dTULP) regulates ciliary localization of TRPs. dTULP-deficient flies show uncoordinated movement and complete loss of sound-evoked action potentials. Inactive and NompC are mislocalized in the cilia of auditory receptor neurons in the *dTulp* mutants, indicating that dTULP is required for proper cilia membrane protein localization. This is the first demonstration that dTULP regulates TRP channel localization in cilia, and suggests that dTULP is a protein that regulates ciliary neurosensory functions.

## Author Summary

Tubby is a member of the Tubby-like protein (TULP) family. *Tubby* mutations in mice (tubby mice) cause late-onset obesity and neurosensory deficits such as retinal degeneration and hearing loss. However, the exact molecular mechanism of Tubby has not been determined. Here we show that *Drosophila* Tubby homolog, King tubby (dTULP), regulates ciliary localization of transient receptor potential protein (TRP). dTULP-deficient flies showed uncoordinated movement and complete loss of sound-evoked action potentials. dTULP was localized in the cilia of chordotonal neurons of Johnston's organ. Two TRP channels essential for auditory transduction, Inactive and NompC, were mislocalized in the cilia of chordotonal neurons in the *dTulp* mutants, indicating that dTULP is required for proper cilia membrane protein localization. This is the first demonstration that dTULP regulates TRP channel localization in cilia, and thus provides novel insights into the pathogenic mechanism of tubby mice.

## Introduction

The auditory system allows animals to communicate and obtain information about their environment. The hearing organs transform sound into an electrical signal through a process called mechanotransduction, the conversion of a mechanical force impinging on a cell into an intracellular signal [1]. Although the recent discovery of several molecules involved in mechanotransduction allows interpretation of the biophysical properties of the mechanotransduction process for hearing [2], many additional molecular players in auditory development and function are waiting to be unveiled.

*Drosophila melanogaster* has been suggested as a model organism to study the fundamental process of hearing [3], [4]. Hearing in the fly is necessary for the detection of courtship songs [5]–[7]. Male-generated courtship song causes females to reduce locomotion and enhances female receptivity, whereas it causes males to chase each other [8]. The ability to hear courtship songs is ascribed to Johnston's organ (JO) in the second antennal segment. Near-field sounds rotate the sound receiver; the third antennal segment and the arista and this rotation of the antennal receiver transmits mechanical forces to the JO in the second antennal segment, which is connected to the third antennal segment by a thin stalk [9]. Each JO sensilla consists of two or three chordotonal neurons and several supporting cells. The outer dendritic segments of the JO neurons are compartmentalized cilia which are directly connected to the antennal sound receiver via extracellular caps. The distortion of the junction between the second and third segment stretches the cilia and stimulates the JO neurons.

Several transient receptor potential (TRP) channels have been shown to be required for *Drosophila* hearing transduction and amplification [4], [10]–[14]. Mutation in *nompC*, the *Drosophila* TRPN channel, resulted in substantial reduction of sound-evoked potentials [4]. Reports showing that NompC and TRP-4 (the *C. elegans* ortholog of NompC) are *bona fide* mechanotransduction channels support the idea that NompC is the *Drosophila* hearing transducer [15], [16]. Two *Drosophila* TRPV channel, *inactive* (*iav*) and *nanchung* (*nan*), mutants showed complete loss of sound-evoked action potentials [11]. However, they have not been considered to be the hearing transduction complex per se; rather they are thought to be required to amplify the electric signal generated by the hearing transduction complex, since Iav and Nan reside in the proximal cilia which are distant from the distal cilia where NompC is localized and mechanical force is directly transmitted [17], [18]. A recent study which employed a new method to measure subthreshold signals from the JO neurons suggested the opposite possibility that the TRPV (Iav and Nan) complex is the hearing transduction complex modulated by TRPN (NompC) [14]. Although the exact roles of each TRP in *Drosophila* hearing are still controversial, it is clear that TRPN and TRPV have essential and distinct roles in *Drosophila* hearing.

Several attempts have been made to identify molecular players regulating the function of the ciliated mechanoreceptor neurons. Gene expression profiling identified chordotonal organ-enriched genes from campaniform mechanoreceptors, developing embryo chordotonal neurons, and the second antennal segment [19]–[21]. Alternatively, chordotonal neuron-specific genes were identified by searching for regulatory factor X (RFX)-binding sites, because ciliogenesis of the chordotonal neurons mainly depends on the RFX transcription factor [22]. However, so far only a limited number of genes involved in TRP channel localization in the JO neuron cilia have been identified and characterized, including axonemal components and intraflagellar transports (IFTs) [17], [23]. IFTs are indispensable for the formation and maintenance of cilia as well as for the transport of proteins along the microtubules in and out of the cilia [24]–[26]. Therefore, mutation of many of the characterized genes results in not only delocalization of the TRPs but also profound structural abnormality in cilia, rendering it difficult to delineate the gene functions specific to TRP localization.

Tubby is the founding member of Tubby-like proteins (TULPs) [27]. Loss-of-function of the *Tubby* gene exhibits adult-onset obesity, retinal degeneration, and hearing loss in mice. The *Drosophila* genome encodes one Tubby homolog called King tubby (hereafter designated dTULP), which shares approximately 43% amino acid identity with mouse Tubby (Figure S1A) [28]. At the embryonic stage, dTULP is expressed in various types of neuronal cells including the chordotonal neurons. Although previous expression analyses and bioinformatic approaches detected *dTulp* in the chordotonal organs, its presence did not attract much interest because of its distribution in various neuronal cell types [22].

In this study, we aimed to investigate the novel molecular function of dTULP in *Drosophila* hearing. dTULP is localized to the well-defined ciliary structure of *Drosophila* auditory organs. Loss of dTULP has no effect on the ciliary structure of the JO neurons, but NompC and Iav localization in cilia was severely altered. These data demonstrate a new role of dTULP as a regulator of TRP localization in the hearing organs.

## Results

### Generation of *dTulp* mutants

**Figure 1:**
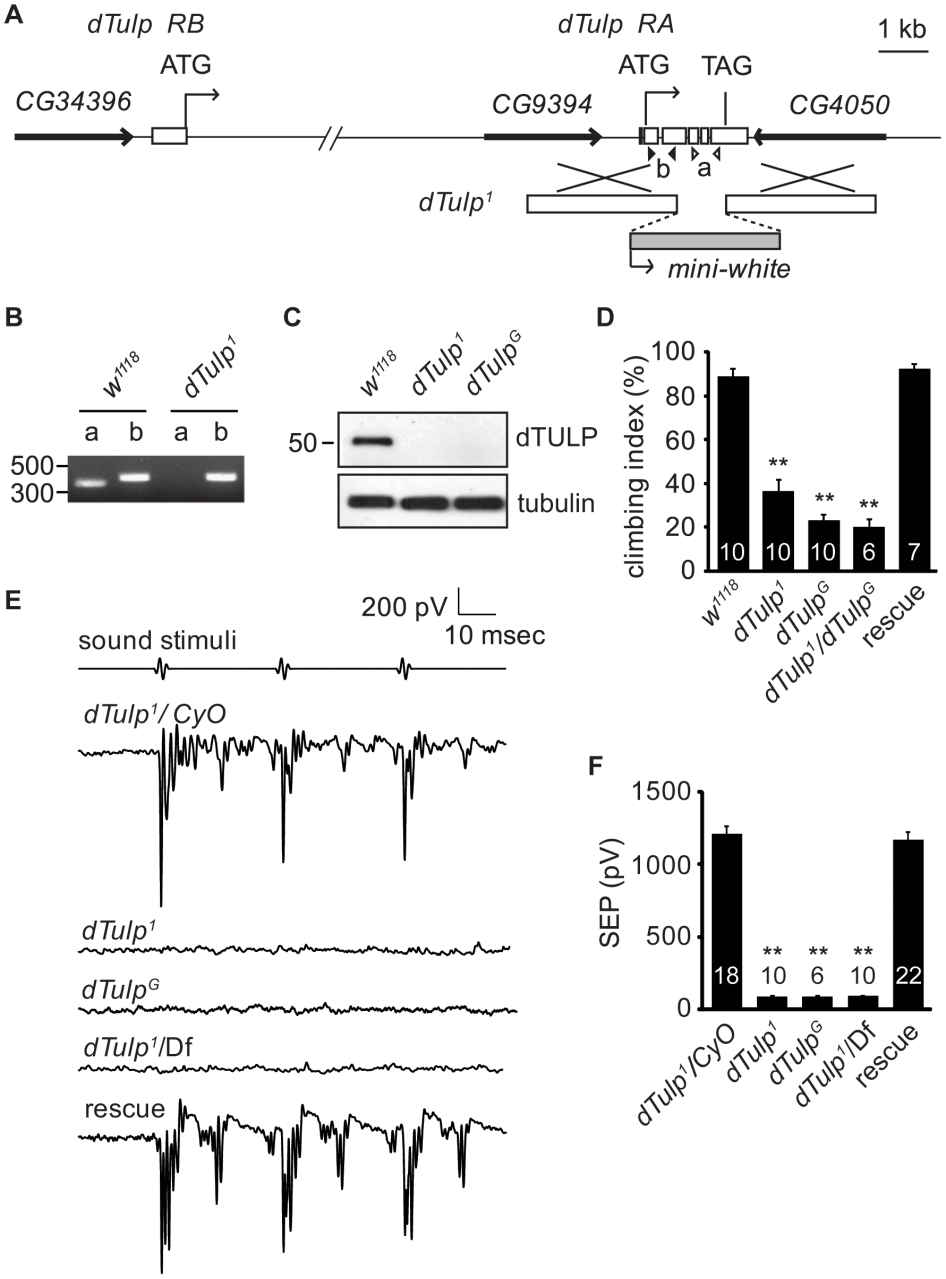
Hearing loss in the *dTulp* mutant. (A) Physical map of the *dTulp* locus and the targeting schemes used to generate the *dTulp^1^* allele by homologous recombination. The boxes indicate *dTulp* exons. Open (a) and solid triangles (b) indicate the locations of primers used for genomic PCR to confirm *dTulp* deletion. (B) Confirmation of the deletion of the *dTulp* locus using genomic PCR. (C) Western blot analyses using extracts from wild-type, *dTulp^1^*, and *dTulp^G^* flies. (D) dTULP-deficient flies exhibit climbing defects. A single insertion of the genomic rescue construct rescues the *dTulp^1^*climbing defect. The number of tests for each genotype is indicated inside the bars. **p<0.01 compared with wild type (*w^1118^*) using one-way ANOVA with *post-hoc* analysis (Tukey). (E) Representative traces of the sound-evoked potentials recorded from the antennal nerve of the indicated genotypes. Df indicates Df(2R)BSC462. The hearing defect was rescued by introduction of a genomic DNA fragment. (F) Quantification of the sound-evoked potentials. Genotypes are indicated. The number of flies used for quantification for each genotype is indicated. **p<0.01 compared to *dTulp^1^/CyO*. *p* values were calculated using ANOVA with *post-hoc* Tukey assay. All error bars indicate SEM.

To test whether dTULP plays a role in *Drosophila* hearing, we generated two *dTulp* mutant alleles by ends-out homologous recombination [29]. The first allele was *dTulp^1^*, which harbours a deleted C-terminal containing the conserved “tubby domain” (residues 220 to 460; Figure 1A). The second allele, *dTulp^G^*, was generated by replacing an N-terminal portion of the dTULP coding region (residues 18 to 261; Figure S1B) with *GAL4* coding sequences at the site corresponding to the initiation codon of the short splicing variant of *dTulp*. Genomic PCR analyses showed that the *dTulp* genomic locus was deleted in *dTulp^1^* and *dTulp^G^* flies (Figure 1B and Figure S1B). We raised antibodies to dTULP, which recognized a 51 kDa protein as predicted in wild-type fly extracts on a Western blot, and confirmed that dTULP was not detected in *dTulp^1^* and *dTulp^G^* fly extracts (Figure 1C). Both alleles are homozygous viable and fertile.

### Hearing impairment in *dTulp* mutant flies

Since both *dTulp^1^*and *dTulp^G^* mutant alleles showed postural problems and uncoordinated movement, we performed a climbing assay. Flies were banged down to the bottom of a vertical tube and the percentage of the flies climbing above half of the height of the vertical tube within 10 seconds was recorded as the climbing index. *dTulp^1^*, *dTulp^G^*, and transheterozygote flies exhibited a decreased climbing index compared to control flies (Figure 1D). Introduction of a P[acman] clone containing the dTULP coding region (CH321-59C17) in the *dTulp^1^* mutant background rescued this phenotype [30]. These data suggested that *dTulp* mutants may have functional defects in the JO neurons [13].

To check for hearing defects in *dTulp* mutant flies, we recorded extracellular sound-evoked potentials in wild-type and dTULP-deficient flies. Sound-evoked potentials were completely abolished in *dTulp^1^*, *dTulp^G^*, and *dTulp^1^* in *trans* with a deletion that completely removed *dTulp, Df*(2R)BSC462. Genomic rescue using the P[acman] clone produced sound-evoked potentials similar to those in the wild-type, suggesting that the hearing defect was specifically due to *dTulp* ablation (Figure 1E and 1F).

### Expression of dTULP in the chordotonal neurons

To test whether dTULP is expressed in the JO neurons, we first attempted to take advantage of the *GAL4*/*UAS* system using the *dTulp^G^* allele. However, the *GAL4* reporter inserted in *dTulp^G^* was not expressed. This may be caused by inserting *GAL4* at the site corresponding to the initiation codon of the short splicing variant of *dTulp* rather than the long splicing variant.

**Figure 2:**
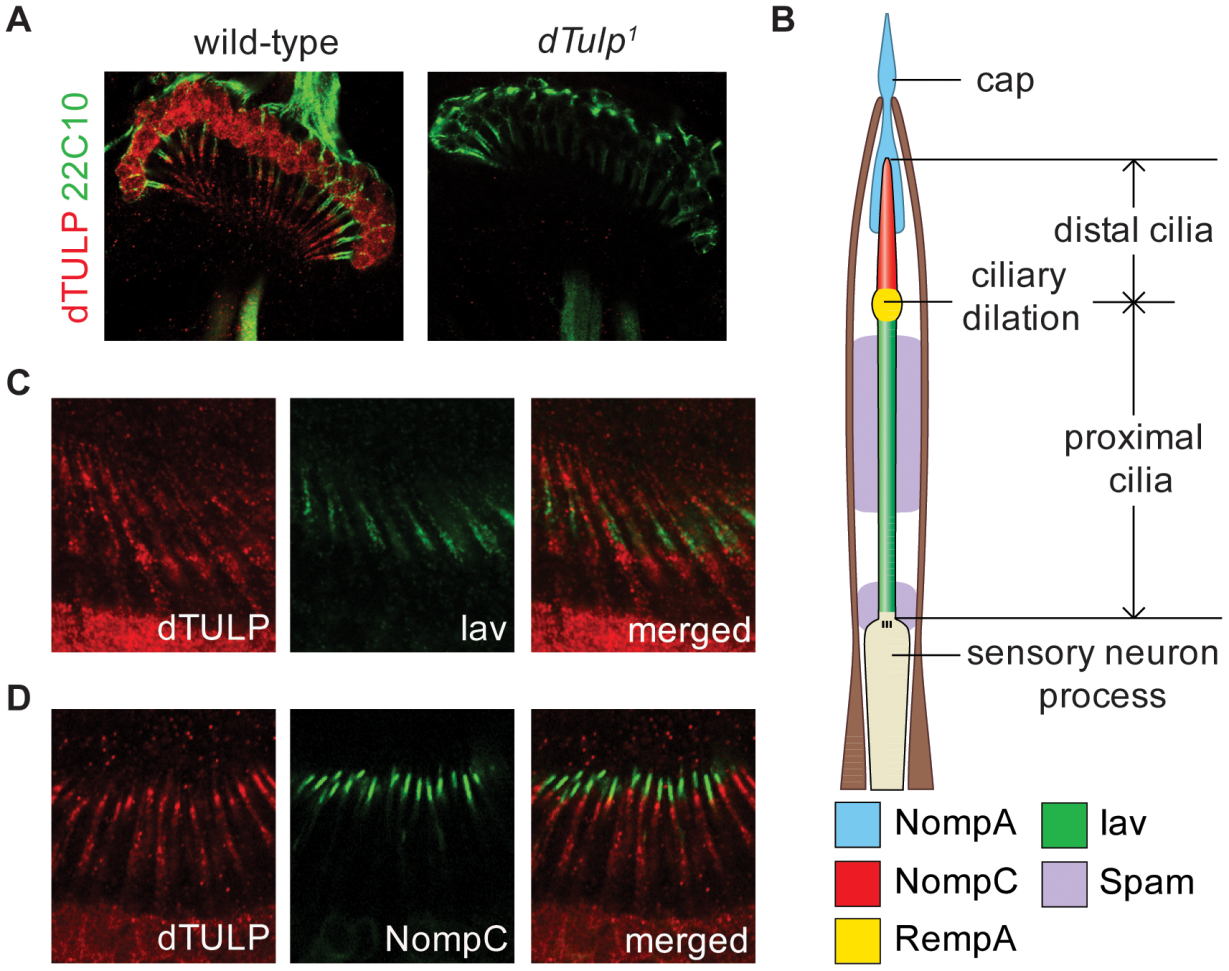
Ciliary localization of dTULP in Johnston's organ. (A) Expression of dTULP in the chordotonal neurons of the Johnston's organ in wild type (left) and *dTulp^1^* (right). 22C10 is a monoclonal antibody that stains all chordotonal neurons except for the cilia. (B) Schematic diagram of the scolopidium in the Johnston's organ. For simplicity, only one sensory neuron process is depicted. The localization of molecular components is marked with shaded colors. (C–D) dTULP localization was analyzed in comparison with two ciliary proteins, Iav and NompC. (C) Immunostaining of dTULP and Iav–GFP (anti-GFP) on the antennae of *Iav*-*GFP* flies. (D) Immunostaining of dTULP and NompC-GFP (anti-GFP) on the antennae of *F*>*UAS-NompC:GFP* flies.

Therefore, we performed immunohistochemistry with dTULP antibodies. We found that dTULP was expressed in the cilia as well as the cell body of the chordotonal neurons (Figure 2A, left). We did not detect dTULP immunoreactivity in the JO neurons in *dTulp^1^*flies, indicating that the immunosignal is specific for dTULP (Figure 2A, right). To further characterize the ciliary localization of dTULP, we compared the localization of dTULP with that of Iav and NompC. The subcellular localization of Iav and NompC are in the proximal and distal cilia, respectively, in a mutually exclusive manner (Figure 2B) [11], [17], [18], [31]. dTULP staining extended from the proximal to distal cilia with a much weaker signal observed in the distal portion (Figure 2C and 2D). The mouse Tubby protein has been reported to shuttle from the plasma membrane to the nucleus upon Gq-coupled G protein-coupled receptor (GPCR) activation [32]. dTULP was also detected in the cell body as well as the nucleus in the JO neurons (Figure 2A and Figure S7B). We also found that dTULP was expressed in other types of sensory neurons with cilia (Figure S2).

### Normal scolopidia structure in *dTulp* mutants

**Figure 3:**
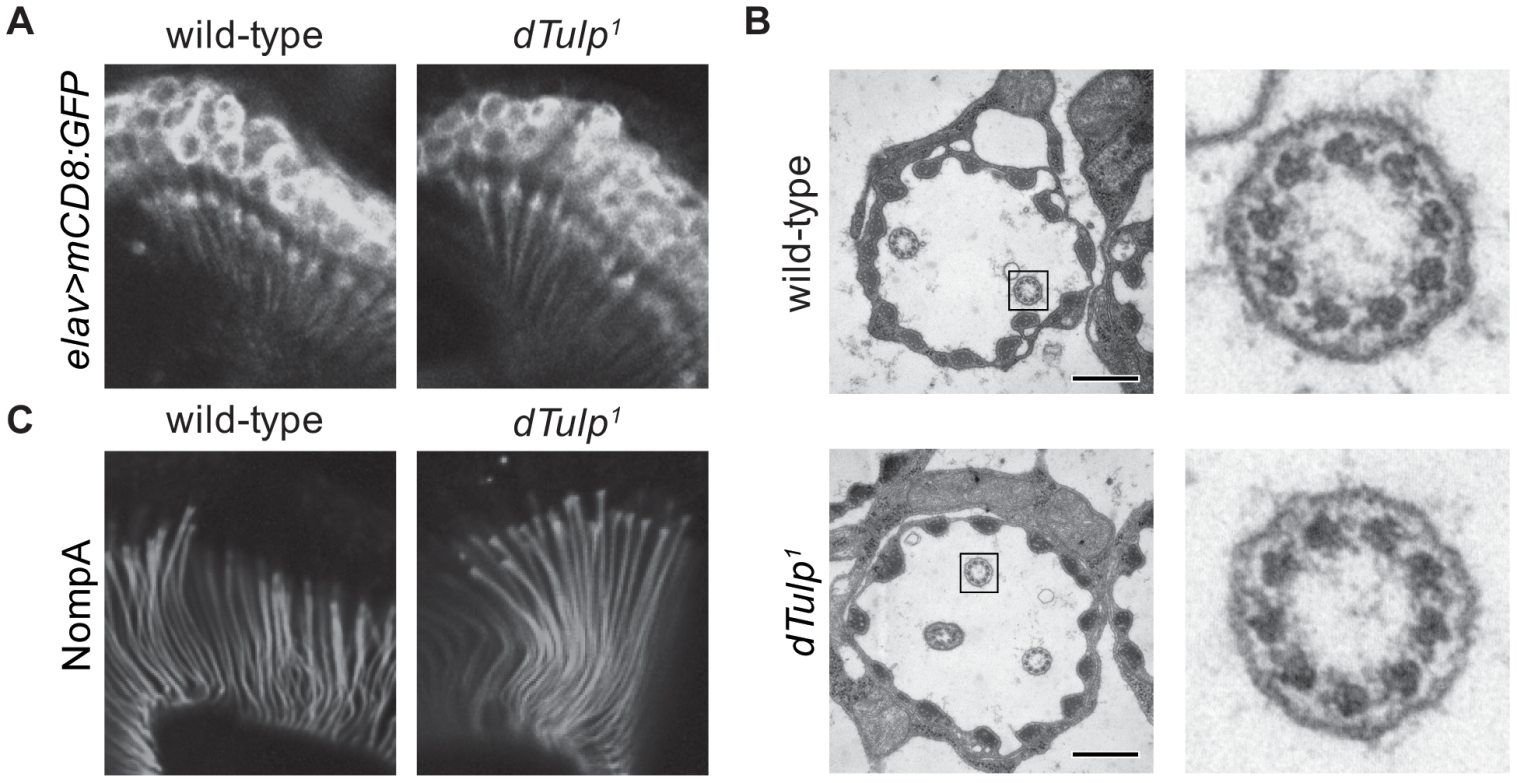
Normal scolopidia structure in the *dTulp* mutant. (A) Confocal images of the second antennal segment expressing mCD8:GFP. Genotypes of animals are *dTulp^1^*/+;e*lav*-*GAL4*/*UAS*-*mCD8:GFP* (left) and *dTulp^1^*/*dTulp^1^*;e*lav*-*GAL4*/*UAS*-*mCD8:GFP* (right). (B) Transmission electron microscopic examination of wild-type and *dTulp^1^*mutant antennal scolopidium. Scale bars represent 0.2 µm. The black boxes mark the inset fields shown at higher magnification on the right. (C) Confocal images of second segment antennae from pupae expressing GFP-tagged NompA. Genotypes of animals are *dTulp^1^*/+;*NompA-GFP*/+ (left) and *dTulp^1^*/*dTulp^1^*;*NompA-GFP*/+ (right).

To examine whether the *dTulp* mutants have developmental defects in the JO neuron structure, we observed the expression of a membrane-targeted GFP (*UAS*-*mCD8:GFP*) driven by the pan-neuronal promoter (e*lav*-*GAL4*) in the JO neurons. We found no gross structural abnormalities in *dTulp^1^*flies (Figure 3A). Electron microscopy of the JO revealed that most *dTulp* mutants had normal ciliary ultrastructure (Figure 3B). Approximately 9.3% of chordotonal scolopidia appeared abnormal in terms of cilia number or cap-cilia connections (Figure S3). In addition, we did not observe any discernible changes in the expression of the dendritic cap protein NompA, which transmits mechanical stimuli to the distal segment of chordotonal neurons in *dTulp* mutants (Figure 3C) [33]. These observations suggest that structural changes in the JO cannot account for severe hearing impairment in *dTulp* mutants.

### Requirement of dTULP for proper ciliary localization of Iav, NompC, and Spam

Mutations of *trps*, including *iav* and *nompC*, cause hearing defects in *Drosophila* [4], [11]. To investigate the possibility that dTULP controls the expression of TRPs and other genes which are indispensable for *Drosophila* hearing, we performed quantitative PCR analysis of such genes and no significant differences in expression levels were present between wild-type and *dTulp^1^* antennae (Figure S4). This suggested that dTULP plays other roles in *Drosophila* chordotonal neurons rather than as a transcription factor that controls transcription of known hearing related genes, although we cannot exclude the possibility that dTULP regulates the expression of hearing related genes we did not survey.

**Figure 4:**
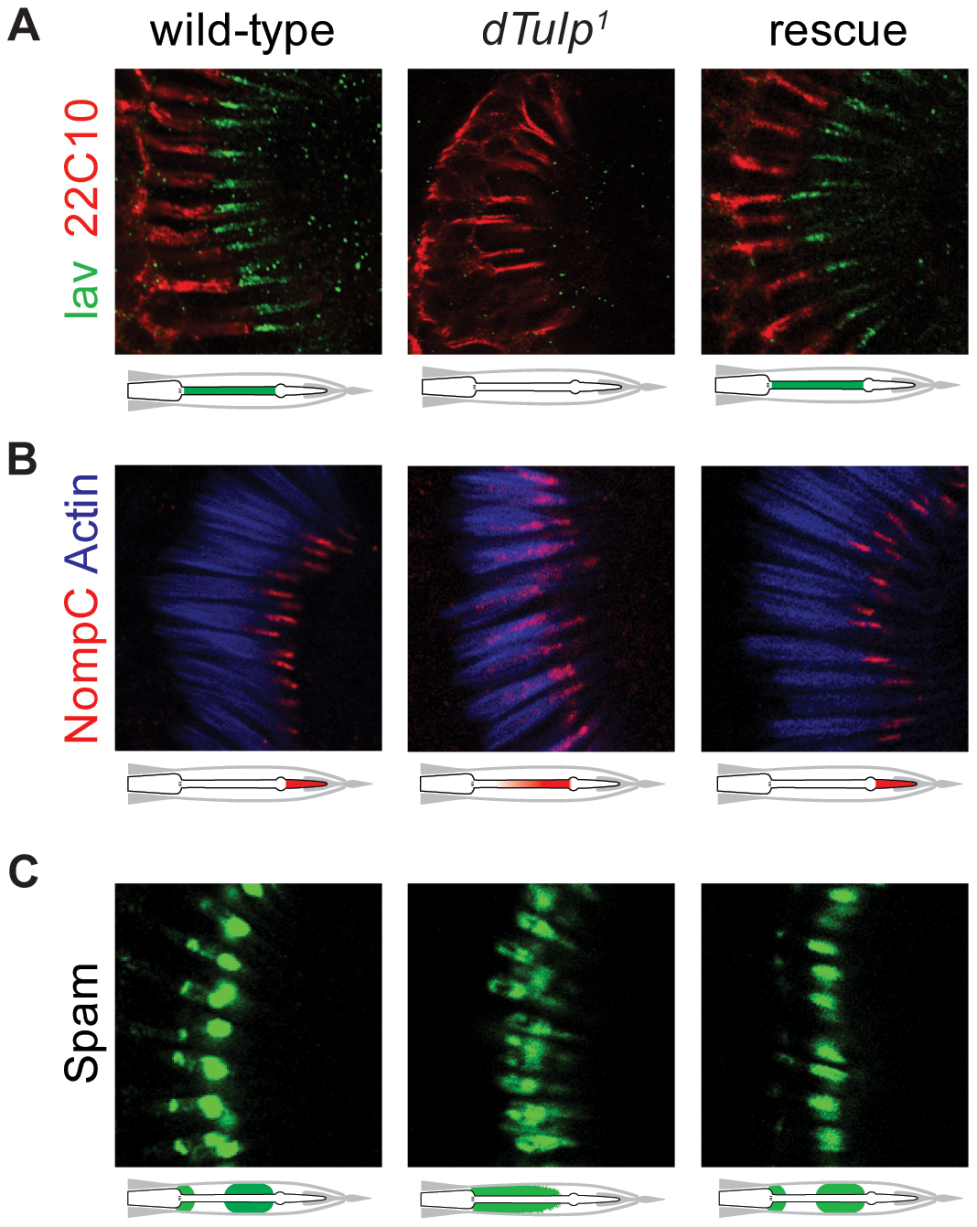
Mislocalization of Iav, NompC and Spam in the *dTulp* mutant. (A–C) Confocal imaging of the second antennal segment from wild-type, *dTulp^1^*, and genomic rescue flies. The localization of Iav-GFP and NompC are depicted by schematic diagrams. (A) Immunostaining of Iav-GFP (anti-GFP) counterstained with 22C10 which stains neuronal cells except for the outer segments, i.e. cilia. (B) Immunostaining of NompC counterstained with phalloidin that specifically stains actin-rich scolopales. (C) Immunostaining of Spam detected with the monoclonal antibody 21A6.

Next we examined the ciliary localization of Iav and NompC in the *dTulp* mutants. Surprisingly, Iav was not localized to the proximal cilia in *dTulp^1^* flies (Figure 4A). Furthermore, NompC, characteristically localized to the distal cilia (Figure 2B), was redistributed toward the proximal cilia (Figure 4B). Spacemaker (Spam) is an extracellular protein which protects cells from massive osmotic stress [34]. Localization of Spam was also altered in *dTulp* mutants from its two typical locations: the luminal space adjacent to the cilia dilation and the scolopidium base (Figure 4C) [35]. Introduction of the *dTulp*+ transgene rescued the localization of Iav, NompC, and Spam (Figure 4).

IFTs are involved in the localization of Iav, NompC, and Spam [17], [23]. Because IFT mutants show similar phenotypes to the *dTulp* mutant, we investigated the localization of IFT proteins in dTULP-deficient flies. Ciliary localization of the two IFTs, NompB (the ortholog of human IFT-B, IFT88) and RempA (the ortholog of human IFT-A, IFT140), was unaffected in *dTulp^1^* mutants (Figure S5).

To further address the functional relationship between dTULP and IFTs, we examined distribution of dTULP in three IFT (*nompB*, *rempA*, and *oseg1*) mutants and a retrograde motor dynein heavy chain (*beethoven*) mutant. Although the *rempA*, *oseg1*, and *beethoven* mutants show different degrees of defective cilia structure, dTULP is localized to the deteriorated cilia of each mutant, suggesting that *rempA*, *Oseg1*, and *beethoven* are not required for the transport of dTULP into the cilia (Figure S6A–S6C). Since the *nompB* mutant does not develop cilia structure, dTULP was present in the inner segment at a high level (Figure S6D) [36]. However, it is possible that other IFTs may play a role for dTULP ciliary localization even though the IFTs we examined are not involved in ciliary localization of dTULP.

### Requirement of both IFT- and phosphoinositide-binding domains of dTULP for the proper cilia localization of TRPs

**Figure 5:**
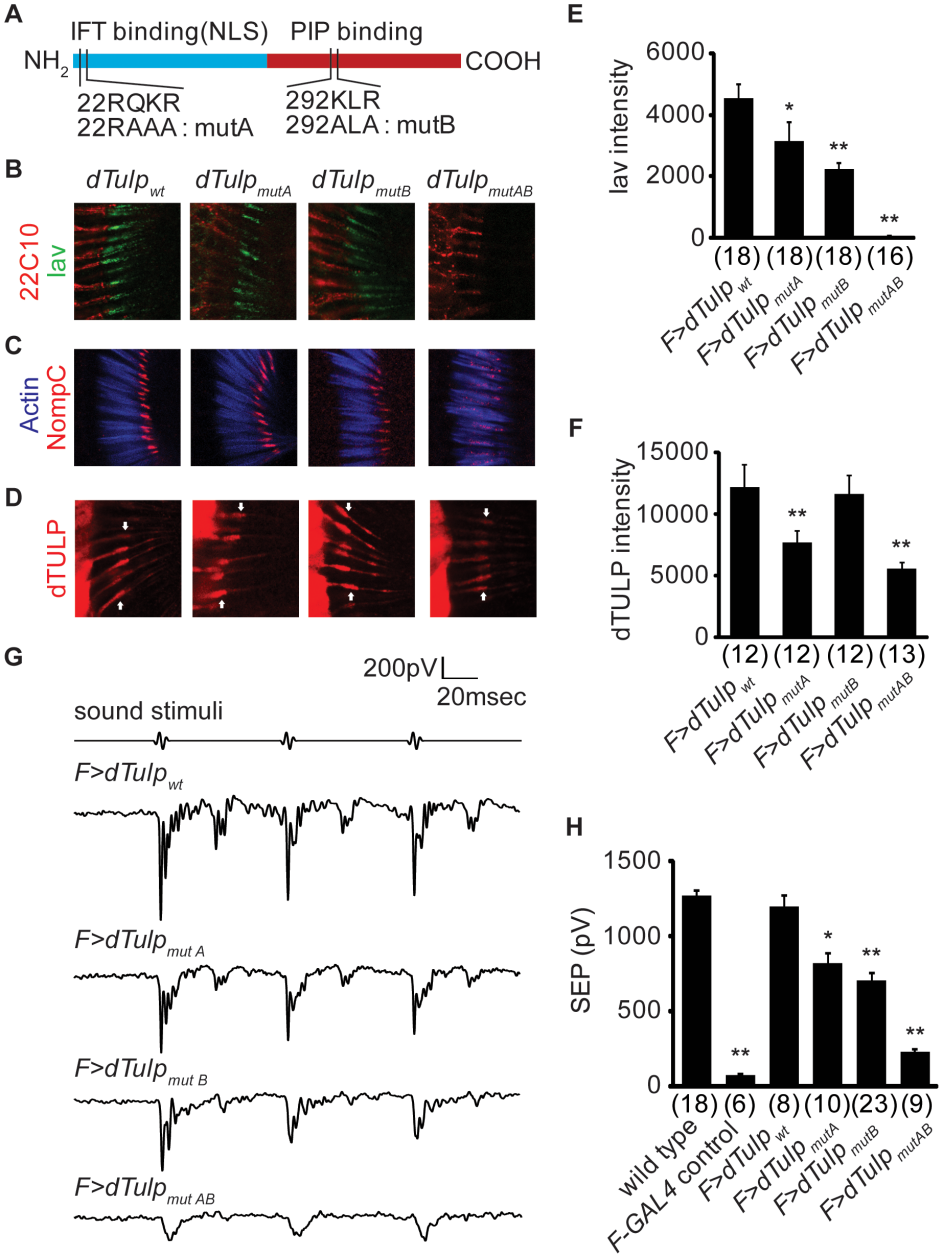
Functional domain mapping of dTULP for ciliary localization of Iav and NompC. (A) Schematic diagram showing different domains of dTULP as well as dTULP mutant forms (mutA, mutB, and mutAB). Location and identity of each mutation are marked. IFT, intraflagellar transport; NLS, nuclear localization signal; PIP, phosphoinositide. (B–D) Confocal imaging of the second antennal segment in the *dTulp* knockout flies expressing dTULP wild-type (dTULP_wt_), dTULP_mutA_, dTULP_mutB_, and dTULP_mutAB_. (B) Confocal imaging of Iav-GFP counterstained with 22C10 which stains neuronal cells except for the cilia located in the outer segment. (C) Immunostaining of NompC counterstained with phalloidin that specifically stains actin-rich scolopales. (D) Immunostaining of dTULP. Arrows indicate the junction between inner and outer segment. (E–F) Quantification of Iav-GFP and dTULP expression levels in the proximal cilia. The number of images analyzed is shown in parentheses. (E) Quantification of Iav-GFP expression level in the proximal cilia. *p<0.05 and **p<0.01 compared to dTULP_wt_-expressing *dTulp* mutant. (F) Quantification of dTULP expression level in the proximal cilia. **p<0.01 compared to dTULP_wt_-expressing *dTulp* mutant. (G) Representative traces of sound-evoked potentials recorded from the antennal nerve of dTULP_wt_, dTULP_mutA_, dTULP_mutB_, and dTULP_mutAB_-expressing *dTulp^1^* flies. (H) Quantification of sound-evoked potentials of indicated genotypes. Genotypes of animal are *dTulp^1^*/*CyO*, *dTulp^1^*,*F-GAL4*/*dTulp^1^*, *dTulp^1^*,*F-GAL4*/*dTulp^1^*;*UAS-dTulp_wt_/*+, *dTulp^1^*,*F-GAL4*/*dTulp^1^*;*UAS-dTulp_mutA_*/+, *dTulp^1^*,*F-GAL4*/*dTulp^1^*;*UAS-dTulp_mutB_*/+, and *dTulp^1^*,*F-GAL4*/*dTulp^1^*;*UAS-dTulp_mutAB_*/+. *p<0.05 and **p<0.01 compared to *dTulp^1^/CyO*. The number of flies used for quantification of each genotype is indicated in parentheses. All *p* values were calculated using ANOVA with *post-hoc* Tukey assay. All error bars represent SEM.

Mammalian Tubby have two distinct domains: nuclear localization signal (NLS) and phosphoinositide (PIP)-binding domain. An NLS, which allows Tubby to translocate into the nucleus, resides in the N-terminal region of Tubby [32]. Recently, a short stretch of amino acids including the NLS in TULP3, a mammalian member of the Tubby-like protein family, has been reported as an IFT-A binding domain [37]. A PIP-binding domain in the C-terminal tubby domain allows Tubby to be localized under the inner leaflet of the plasma membrane through binding to specific phosphoinositides. These domains are also conserved in dTULP (Figure 5A).

In order to investigate the mechanism by which dTULP regulates the ciliary localization of Iav and NompC, we introduced mutations into the putative NLS/IFT-binding (dTULP_mutA_), PIP-binding domain (dTULP_mutB_), or both domains (dTULP_mutAB_) of *dTulp* cDNA and generated *UAS*-*wild-type dTulp (UAS-dTulp_wt_)*, *UAS*-*dTulp_mutA_*, *UAS*-*dTulp_mutB_*, and *UAS*-*dTulp_mutAB_* transgenic flies, respectively. To eliminate positional effects, all transgenes were integrated into the same loci using site-specific recombination with an attP landing site on the third chromosome [38].

To test the effect of each mutation on the subcellular localization of dTULP, we examined the subcellular localization of dTULP_wt_, dTULP_mutA_, and dTULP_mutB_ in *Drosophila* salivary glands. dTULP_wt_ was detected mainly in the plasma membrane and nucleus (Figure S7A). Mutations in the NLS/IFT-binding domain or PIP-binding domain of dTULP resulted in significant exclusion from the nucleus or accumulation in the nucleus, respectively, which suggested that the NLS/IFT-binding and PIP-binding properties of mouse Tubby are conserved in dTULP in *Drosophila* salivary glands (Figure S7A). However, the localization of dTULP_wt_, dTULP_mutA_, and dTULP_mutB_ in the JO neurons in terms of the cell body and nuclear distribution was virtually the same (Figure S7B). These data suggested that dTULP is not shuttled between the plasma membrane and the nucleus in the JO neurons and these domains may have other functions in the JO neurons rather than controlling the translocation of dTULP from the plasma membrane to the nucleus.

To evaluate the functional consequences of each mutation, we expressed dTULP_wt_, dTULP_mutA_, dTULP_mutB_, or dTULP_mutAB_ in the JO neurons of *dTulp^1^* flies. The expression of dTULP_wt_ in the *dTulp* mutant background restored the distribution and the expression level of Iav and NompC similar to those of wild type (Figure 5B and 5C). The expression of dTULP_mutA_ or dTULP_mutB_ rescued the Iav trafficking defect of the *dTulp* mutant, but the expression levels of Iav in the proximal cilia in dTULP_mutA_- or dTULP_mutB_-expressing flies were reduced compared to those of dTULP_wt_-expressing flies (Figure 5B and 5E). NompC localization to the distal cilia in dTULP_mutA_- or dTULP_mutB_-expressing flies was similar to that in dTULP_wt_-expressing flies (Figure 5C). dTULP_mutAB_ could not rescue the Iav or NompC localization defects of the *dTulp* mutant. This difference was not due to the expression levels of the mutant *dTulp* transgene since the expression levels of mutant forms of dTULP were similar to those of wild-type dTULP (Figure S8).

Next, we examined whether the different degrees of rescue of Iav and NompC localization was due to differential ciliary trafficking of variant forms of dTULP. The ciliary expression level of dTULP_mutB_ was similar to that of dTULP_wt_, whereas the ciliary expression levels of dTULP_mutA_ and dTULP_mutAB_ were reduced compared with those of dTULP_wt_ (Figure 5D and 5F). These data suggested that the putative NLS/IFT-binding domain of dTULP has a regulatory function to control the trafficking of dTULP into the cilia.

Consistent with immunohistochemical analyses, dTULP_wt_ fully rescued the hearing defect of the *dTulp* mutant. dTULP_mutA_ and dTULP_mutB_ restored a partial function and dTULP_mutAB_ had no such activity (Figure 5G and 5H).

## Discussion

In the current study, we demonstrate that dTULP is a cilia trafficking regulator in the *Drosophila* hearing system. Mutation of *dTulp* results in hearing loss due to the mislocalization of two TRP channels, Iav and NompC, which are ciliary membrane proteins. In addition, Spam, whose localization is dependent on the IFT machinery, is also mislocalized in *dTulp* mutants.

How does dTULP regulate the ciliary distribution of TRPs in the JO neurons? Several studies have shown that mutations in IFT machinery or cilia components result in mislocalization of Iav, NompC, and Spam, along with abnormal axonemal structure [17], [23]. It is notable that, in contrast to IFT or cilia component mutants, ciliogenesis and maintenance appear normal in dTULP-missing flies. Furthermore, the altered distribution of Iav, NompC, and Spam in *dTulp* mutants was not due to the mislocalization of IFT proteins, since the localization of two IFTs (NompB and RempA) was normal in *dTulp* mutants (Figure S6). These data suggest that dTULP acts downstream of the IFTs to regulate TRP localization.

Even though the mutation of *dTulp* affected the trafficking of both Iav and NompC, the compartmentalized ciliary localization of Iav and NompC is differentially regulated by dTULP. An individual mutation in either the putative IFT- or PIP-binding domain reduced Iav expression levels in cilia, whereas NompC localization was not altered until both domains were mutated. Even after the double mutations in both domains of dTULP, NompC is still situated inside the cilia, but in abnormal locations. These findings demonstrate that ciliary entry of NompC is not dependent on dTULP while the distal ciliary localization of NompC is dependent on dTULP. One possibility is that dTULP allows NompC to disengage from the IFT complex at the distal cilia so that NompC is enriched in the distal cilia through the mechanism that required both IFT- and PIP-binding domains. It is also possible that the distal ciliary localization of NompC is regulated by an unidentified factor(s) whose ciliary localization is dTULP-dependent as is Iav.

Both the putative IFT- and PIP-binding domains play important roles in the proper Iav distribution in cilia, but they appear to have different roles. Even though the IFT- or PIP-binding mutant forms of dTULP could only partially rescue the ciliary levels of Iav to the similar extent, the mutation of the IFT-binding domain reduced the ciliary levels of dTULP while disruption of the PIP-binding domain had no effect on the ciliary levels of dTULP. These findings suggest that two domains play distinct roles in the regulation of the ciliary localization of Iav. The IFT-binding domain is the motif required for the ciliary entry for dTULP, and the PIP-binding domain is not related to dTULP ciliary entry itself, rather it affects recruitment of Iav-containing preciliary vesicles to dTULP. By these two linked steps, Iav localization to cilia would be facilitated by dTULP.

In mammals, IFT-A directs the ciliary localization of TULP3 through physical interaction between TULP3 and the IFT-A core complex (WDR19, IFT122, and IFT140), and in turn, promotes trafficking of GPCR to the cilia. Indeed, the depletion of individual IFT-A core complex components affects the ciliary localization of TULP3, which results in the inhibition of GPCR trafficking to the cilia [37]. It appears that dTULP and TULP3 have the similar molecular mechanisms to regulate ciliary membrane proteins. However, unlike TULP3, dTULP ciliary access is not dependent on IFT-A. dTULP ciliary trafficking was not affected by the mutation of *Oseg1* (an ortholog of human IFT-A, IFT122) or *rempA* (an ortholog of human IFT-A, IFT140). Furthermore, the presence of dTULP in cilia did not determine the normal localization of Iav. For example, in the *rempA* mutant, even when dTULP was localized to the cilia (Figure S6B), Iav was not found in cilia [23]. Taken together, dTULP facilitates the relay of preciliary vesicles to the IFT complex at the base of cilia rather than moving together with ciliary membrane proteins into the cilia as an adaptor between IFT and cargo. dTULP may have other additional roles in cilia, which needs to be explored in the future. Based on our finding that dTULP but not Iav could be found in cilia of IFT mutants, it is also possible that recruitment of Iav-containing preciliary vesicles requires dTULP and additional unknown factors, whose function is altered in IFT mutants. Thus, Iav-containing preciliary vesicles may not be able to form stable interactions with dTULP and IFTs.

After the cloning of the *Tubby* gene two decades ago, one promising hypothesis has been that Tubby is a transcription factor, since Tubby translocates to the nucleus upon GPCR activation and the N-terminal region of Tubby has transactivation potentials [32], [39]. However, candidate target genes for Tubby have not been identified. Tubby is thought to have additional functions including vesicular trafficking, insulin signaling, endocytosis, or phagocytosis [40]–[43]. It is still not clear how these molecular functions lead to the *in vivo* phenotypes observed in the tubby mouse. Meanwhile, several studies have hinted at possible connections between the phenotypes of *tubby* mutant mice and ciliary dysfunction. Tubby mice phenotypes comprise syndromic manifestations that are commonly observed in ciliopathies such as Bardet-Biedle syndrome [44] and Usher syndrome [45], [46]. Recently, GPCR trafficking into neuronal cilia was reported to be misregulated in tubby mice [47]. Mutation of *Tulp1*, a member of the TULPs, in human and mice, exhibits retinal degeneration due to the mislocalization of rhodopsin [48]. TULP3 represses Hedgehog signalling, which is a crucial signalling cascade in cilia, via the regulation of the ciliary localization of GPCRs [49]. Our current study provides additional supports for the idea that TULPs play an important role in ciliary signalling and that the tubby mouse syndrome might be due to the ciliary defects.

In contrast to mammalian cells, only specialized cell types have the ciliary structure in *Drosophila*, and the expression of dTULP is not restricted to organs with the ciliary structure, which suggested that dTULP may have other roles not related to the ciliary function [28]. For example, dTULP mediates rhodopsin endocytosis in *Drosophila* photoreceptor cells which do not have cilium in contrast to its mammalian counterpart [50].

In summary, we demonstrate an intriguing role of dTULP in governing the ciliary localization of TRP proteins. This is the first *in vivo* evidence showing that dTULP may have important roles in the maintenance of ciliary functions by regulating the localization of ciliary proteins, thereby maintaining sensory functions.

## Materials and Methods

### Fly stocks

All fly stocks were maintained in regular laboratory conditions (conventional cornmeal agar molasses medium, 12-h light/12-h dark cycle at 25°C, 60% humidity). *Iav*-*GFP* and *NompA*-*GFP* were reported previously [13], [33]. *RempA*-*YFP* and *NompB*-*GFP* were from M. Kernan. Y. Jan and M. Noll provided *UAS*-*NompC:GFP* and *Poxn*-*GAL4*, respectively. Df(2R)BSC462, e*lav*-*GAL4*, *UAS*-*mCD8:GFP*, *AB1-GAL4*, *F-GAL4*, and *Orco-GAL4* were from the Bloomington Stock Center (Bloomington, IN).

### Generation of *dTulp* mutant flies

We employed ends-out homologous recombination to generate *dTulp* mutant alleles. To make the *dTulp^1^* allele, 3 kb genomic DNA at the 5′ and 3′ ends of the tubby domain (220 to 460 residues) coding sequence was PCR amplified from *w^1118^* and subcloned into the pw35 vector. The primer sequences for the 5′ homologous arm of the pw35 vector are 5′-AAAGCGGCCGCCACCGGTGACATCCTCATGTTC-3′ and 5′-AAAGCGGCCGCGTTGCATCACGAACTGGTCGATATTG-3′. The primer sequences for the 3′ homologous arm of the pw35 vector are 5′-TGAGCTGGCTGGGATCCTCGGGTTGG-3′ and 5′-GTGGATCCTTCCTGGTTGGCATCACGTTGAC-3′. To generate the *dTulp^G^* allele, we used the pw35*GAL4loxP* vector in which *GAL4* and *white* are flanked by loxP sequences so the cassette can be removed by introducing Cre recombinase. We subcloned the 3 kb of genomic DNA from each of the 5′ and 3′ ends of the dTULP coding region (18 to 261 residues) into the pw35*GAL4loxP* vector. The primer sequences for the 5′ homologous arm of the pw35*GAL4loxP* vector are5′-ACAGATCTCACCGTCGCCTGGCTCAGTGCCC-3′ and 5′-GTGGTACCCAGCTGGCGCTGCAAAGCAGTTAAATC-3′. The primer sequences for the 3′ homologous arm of the pw35*GAL4loxP* vector are5′-AAAGCGGCCGCGTGGGTTATTGATAGTGATCCTCTA-3′ and 5′-AACCGCGGCGTACAGAATACTCCCTGTTCATGTCT-3′. We generated transgenic flies by germ line transformation (BestGene Inc., Chino Hills, CA) and screened for the targeted alleles as described previously [51]. Targeted alleles were subjected to outcross for five generations into a *w^1118^* genetic background.

### Molecular biology and generation of transgenic flies

We amplified *dTulp* cDNAs from cDNA clones (RE38560) with PCR and subcloned the fragments into the pUASTattB vector. These constructs were subjected to further modification. We generated the *dTulp*_mutA_ and *dTulp_mutB_* mutant constructs using site-directed mutagenesis to change the sequence encoding R23QKR to L23AAA, and K292LR to A292LA, respectively. The dTulp_mutAB_ construct was generated by introducing the mutation corresponding to dTulp_mutB_ into the dTulp_mutA_ construct. To generate genomic rescue transgenic flies, we obtained the BAC clones CH321-59C17 from the BACPAC Resource Center (Oakland, CA) and used these as genomic rescue constructs. Transgenic flies were generated using PhiC31 integrase-mediated transgenesis on the third chromosome to minimize position effect (Bloomington stock number 24749).

### Electrophysiology

Sound-evoked potentials were recorded as described by Eberl et al [4]. Briefly, the fly's antennal sound receivers were stimulated by computer-generated pulse songs. Neuronal responses were detected using a recording electrode inserted in the junction between the first and second antennal segment and a reference electrode was inserted in the dorsal head cuticle. The signals were subtracted with a DAM50 differential amplifier (World Precision Instruments, Sarasota, FL) and digitized using Superscope 3.0 software (GW Instruments, Somerville, MA). Each trace represents the average responses to 10 stimuli.

### Immunohistochemistry

For whole-mount staining, antennae were dissected at the pupa stage and the labellum and legs were prepared at the adult stage. Salivary glands were dissected from third instar larvae. For antenna sections, fly heads were embedded in OCT medium and 14 µm frozen cryostat sections were collected. Dissected tissues and sections were fixed for 15 min with 4% paraformaldehyde in 1× PBS containing 0.2% TritonX-100 (PBS-T) and washed three times with PBS-T. The fixed samples were blocked for 30 min with 5% heat-inactivated goat serum in PBS-T and incubated overnight at 4°C in primary antibodies diluted in the same blocking solution. The tissues were washed three times for 10 min with PBS-T and incubated for 1 h at room temperature in secondary antibodies diluted 1∶500 in blocking solution. Following three washes with PBS-T, the samples were mounted with Vectashield (Vector Laboratories, Burlingame, CA) and examined using a Zeiss LSM710 confocal microscope (Jena, Germany).

To quantify Iav-GFP and dTULP expression levels in cilia, all samples were prepared at the same time and all confocal images were obtained under the same conditions. The pixel intensity of each protein was measured using Zen Software (Jena, Germany). Iav-GFP intensity was measured without immunostaining.

### Antibodies

Rabbit dTULP antibodies were raised by injecting animals with a purified His-tagged dTULP fusion protein (residue 95–339), followed by affinity purification. The primary antibodies were used in immunohistochemistry at the following dilutions: rabbit anti-dTULP, 1∶400; 22C10, 1∶200 (Hybridoma Bank, University of Iowa); 21A6, 1∶200 (Hybridoma Bank); rabbit anti-Orco, 1∶1,000 (gift from L. Vosshall); rabbit anti-NompC, 1∶20; rabbit anti-GFP, 1∶1,000 (Molecular Probes, Eugene, OR); mouse anti-GFP, 1∶500 (Molecular Probes). The secondary antibodies used were Alexa 488-, Alexa 568-, and Alexa 633-conjugated anti-mouse or anti-rabbit IgG (Molecular Probes; 1∶500). DNA and actin were visualized by DAPI and Alexa Fluor 633 Phalloidin (Molecular Probes) staining, respectively.

### Western blot

Fly head or antennae lysates from each genotype were subjected to electrophoresis on SDS-polyacrylamide gels and transferred onto polyvinylidene fluoride membranes. The membranes were blocked for 1 h with 5% nonfat milk plus 0.1% Tween-20. Membrane-bound proteins were analyzed by immunoblotting with primary antibodies against dTULP (1∶1,000) and tubulin (Hybridoma Bank, 1∶2,000).

### Transmission electron microscopy

Fly heads were dissected and fixed in 2% paraformaldehyde, 2.5% glutaraldehyde, 0.1 M cacodylate, and 2 mM CaCl_2_, pH 7.4. The tissue was embedded in LR white resin. Thin sections were cut, mounted on formvar-coated single slot nickel grids, counterstained with uranyl acetate and lead citrate, and examined on a Hitachi H-7500 electron microscope (Hitachi, Tokyo, Japan).

### Real-time PCR

Total RNA was extracted from adult antennae using Trizol reagent (Invitrogen, Carlsbad, CA). cDNA was generated from 0.5 µg of RNA from each genotype using the SuperScript III First Strand Synthesis System (Invitrogen). Quantitative PCR was performed using an ABI7500 real-time PCR machine (Applied Biosystems, Foster City, CA) and the ABI SYBR green system. Transcript levels were normalized to *rp49* as an internal control and the Δ*C_T_* (*C_T_* = threshold cycle) method was used to calculate the relative amount of mRNAs.The primers used for qRT-PCR are listed in Table S1.

### Behavioural test (bang test)

Fifteen 3- to 6-day-old flies were placed in an empty fly food vial. The climbing index is the fraction of flies that climb halfway up the vials in 10 s after being tapped down to the bottom of the tube. We performed each experiment twice and used the average of the two trials to calculate the climbing index.

### Statistical analyses

Data shown are the mean ± SEM. To compare two sets of data, unpaired Student's t-tests were used. ANOVA with the Tukey *post-hoc* test was used to compare multiple sets of data. Asterisks indicate statistical significance.

## Supporting Information

Figure S1 Phylogenetic tree of dTULP and generation of *dTulp^G^* mutant. (A) The phylogenetic relationship between mouse Tubby-like protein family proteins, *C.elegans* Tubby (tub-1), and *Drosophila* King tubby (also known as dTULP). The dendrogram was drawn using TreeDyn. (B) The *dTulp* genomic locus and *dTulp* targeting constructs used to make the *dTulp^G^* allele. PCR primers are depicted by open (a) and solid triangles (b). Genomic PCR of control (*w^1118^*) and *dTulp^G^* flies confirmed deletion of part of the dTULP coding sequence. However, due to lack of GAL4 expression, we could not use the *dTulp^G^* flies as a reporter.
(TIF)

Figure S2 dTULP expression in sensory neurons which have ciliary structure. (A) Coexpression of dTULP in Orco-expressing olfactory receptor neurons. *Orco-GAL4*/*UAS-mCD8:GFP* fly antennae were used for immunostaining. (B) Coexpression of dTULP in labellar gustatory receptor neurons. *Poxn-GAL4*/*UAS-EGFP* fly labella were used for immunostaining. (C) dTULP expression in the femoral chordotonal organ which is marked with *F-GAL4*/*UAS-mCD8:GFP*.
(TIF)

Figure S3 Transmission electron microscopy showing two different types of abnormal scolopidia structure in flies missing dTULP. (A) Abnormal number of chordotonal cilia was observed in 6.2% of examined scolopidia in *dTulp^1^* flies. Arrows indicate ciliary axonemes. (B) The chordotonal cilia were found outside the cap structure in 3.1% of examined scolopidia in *dTulp^1^* flies. Arrows indicate cilia outside the cap.
(TIF)

Figure S4 Comparison of mRNA expression of IFTs and hearing-related genes between wild-type and *dTulp^1^* flies. Antennae extracts from wild-type and *dTulp^1^* flies were used for quantitative PCR analyses. Blue and red bars represent wild type and *dTulp^1^*, respectively (n = 4–7). Error bars indicate SEM. A two-tailed Student's t test was used to test the statistical significance between wild type and *dTulp^1^*.
(TIF)

Figure S5 IFT localization in the *dTulp^1^* mutant. (A) Immunostaining of NompB (anti-GFP) counterstained with 22C10 antibodies on antennae of wild type (*dTulp^1^*/+;*NompB-GFP*/+) and the *dTulp^1^* mutant (*dTulp^1^*/*dTulp^1^*;*NompB-GFP*/+). (B) Confocal imaging of antennae expressing RempA-YFP from wild-type (*dTulp^1^*/+;*RempA-YFP*/+) and *dTulp^1^*(*dTulp^1^*/*dTulp^1^*;*RempA-YFP*/+) flies counterstained with phalloidin.
(TIF)

Figure S6 dTULP localization in IFT mutants. (A–D) Immunostaining of dTULP counterstained with 22C10 antibodies on antennae from indicated IFT-related mutants. (A) Ciliary localization of dTULP in *beethoven* (*btv^5P1^*). (B) Ciliary localization of dTULP in *rempA* (*rempA^1^*). (C) Ciliary localization of dTULP in *oseg1* (*oseg1^EP3616^*). (D) Ciliary localization of dTULP in *nompB* (*nompB^1^*).
(TIF)

Figure S7 Intracellular localization of different mutant forms of dTULP. (A) Confocal imaging of a third instar larval salivary gland expressing dTULP_wt_, dTULP_mutA_, and dTULP_mutB_. Dissected tissues were immunostained using dTULP antibodies and counterstained with DAPI to visualize the nuclei. *AB1*-*GAL4* was used to drive salivary gland expression of each transgenes. (B) Confocal imaging of the second antennal segment expressing dTULP_wt_, dTULP_mutA_, and dTULP_mutB_. Dissected antennae were immunostained using dTULP and ELAV antibodies which labeled neuron nuclei.
(TIF)

Figure S8 Expression level of various forms of dTULP in the second antennal segment. Western blot probed with antibodies to dTULP and tubulin. Samples were prepared from antennae of the indicated flies. Genotypes are wild-type (*w^1118^*), *dTulp^1^,F*-*GAL4*/*dTulp^1^*;*UAS*-*dTulp_wt_*/+, *dTulp^1^,F*-*GAL4*/*dTulp^1^*;*UAS*-*dTulp_mutA_*/+, *dTulp^1^,F*-*GAL4*/*dTulp^1^*;*UAS*-*dTulp_mutB_*/+, *dTulp^1^,F*-*GAL4*/*dTulp^1^*;*UAS*-*dTulp_mutAB_*/+.
(TIF)

Table S1 Sequence information of real-time PCR primers.
(DOCX)
