# Supplementary figures and images for: dTULP, the Drosophila melanogaster Homolog of Tubby, Regulates Transient Receptor Potential Channel Localization in Cilia
Source: PLoS Genet. 2013 Sep 19;9(9):e1003814. doi: 10.1371/journal.pgen.1003814 (PMC3778012; doi:10.1371/journal.pgen.1003814)

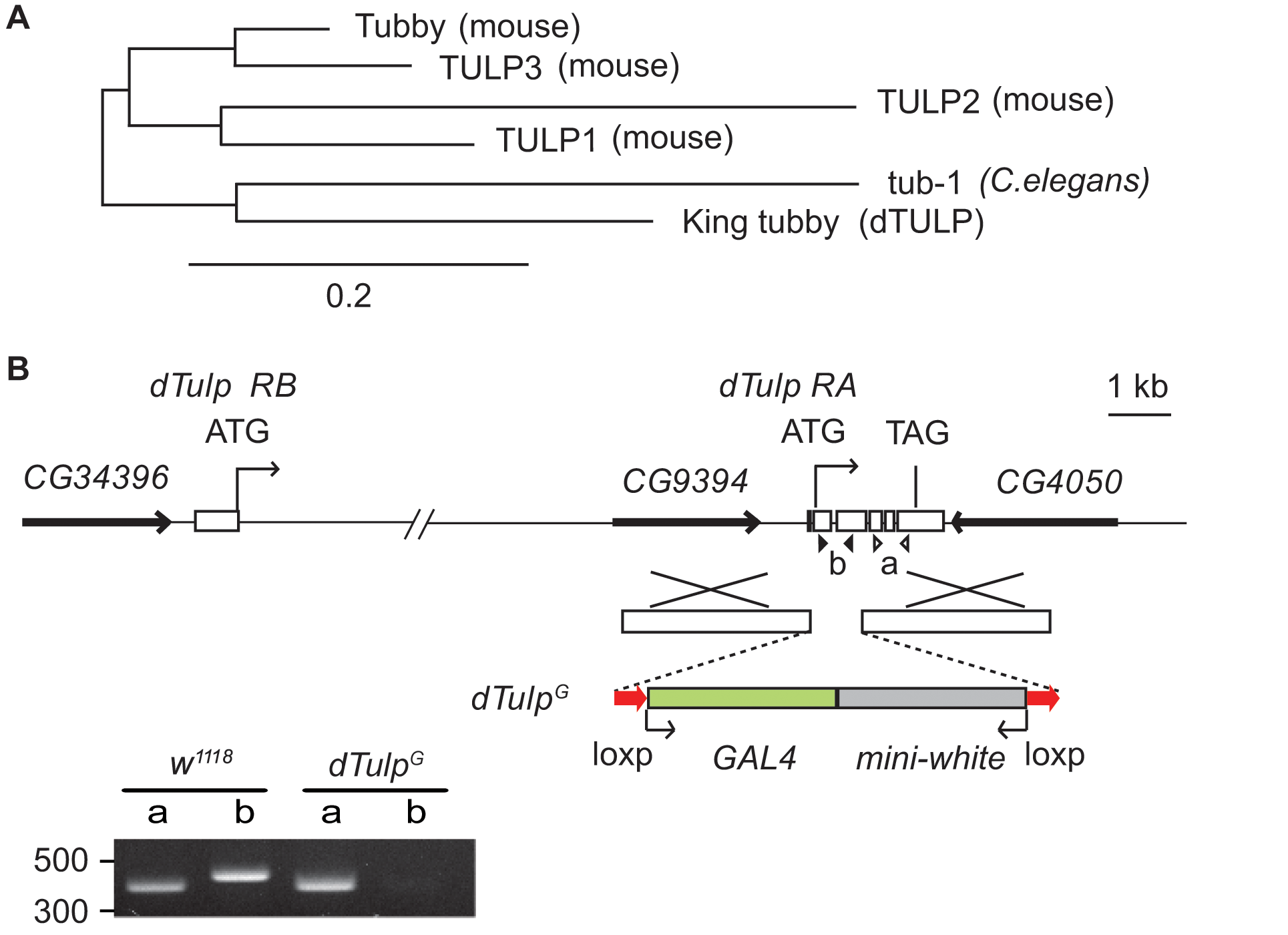

Supplement: Figure S1 — Phylogenetic tree of dTULP and generation of dTulpG mutant. (A) The phylogenetic relationship between mouse Tubby-like protein family proteins, C.elegans Tubby (tub-1), and Drosophila King tubby (also known as dTULP). The dendrogram was drawn using TreeDyn. (B) The dTulp genomic locus and dTulp targeting constructs used to make the dTulpG allele. PCR primers are depicted by open (a) and solid triangles (b). Genomic PCR of control (w1118) and dTulpG flies confirmed deletion of part of the dTULP coding sequence. However, due to lack of GAL4 expression, we could not use the dTulpG flies as a reporter. (TIF) [file pgen.1003814.s001.tif]

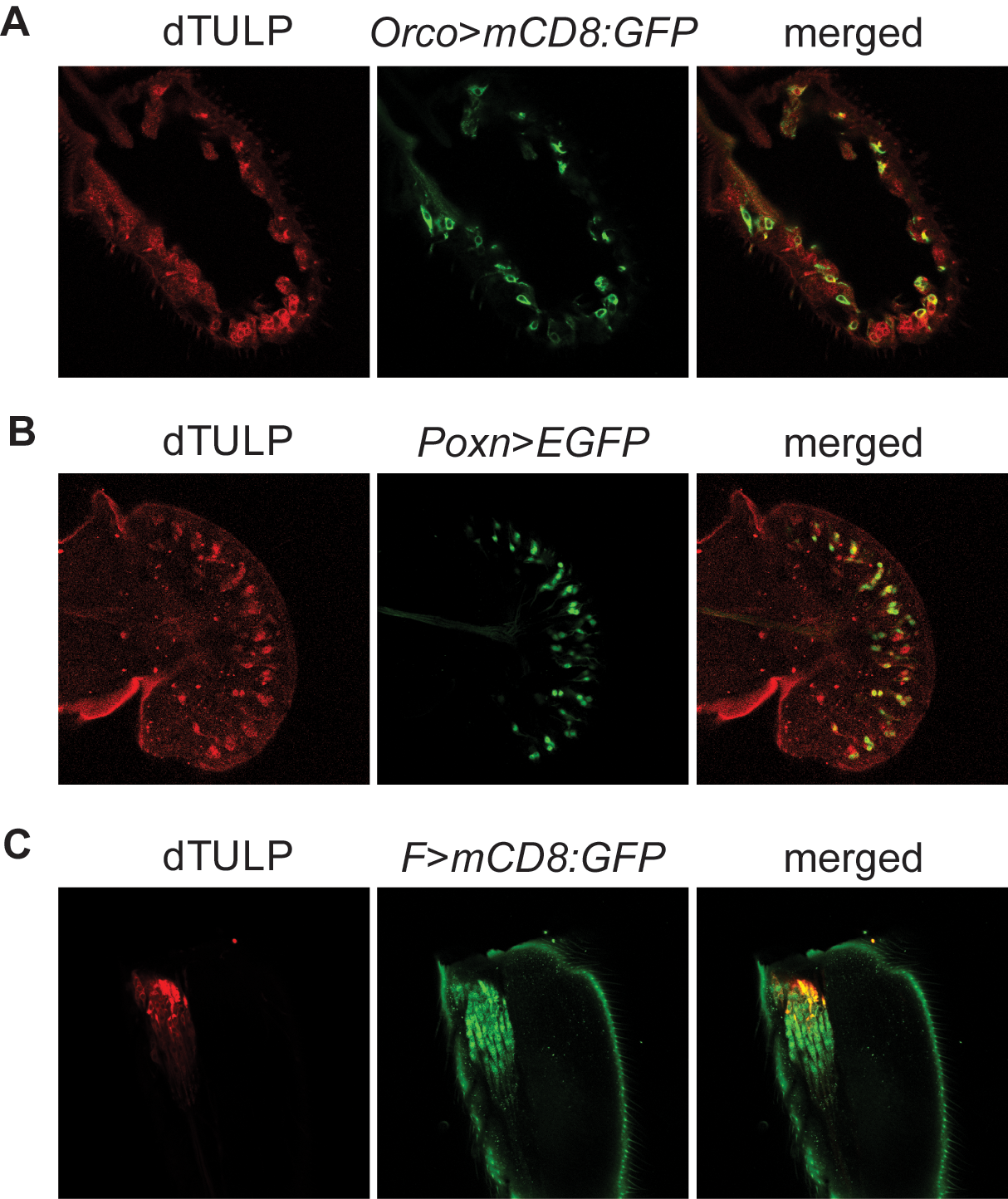

Supplement: Figure S2 — dTULP expression in sensory neurons which have ciliary structure. (A) Coexpression of dTULP in Orco-expressing olfactory receptor neurons. Orco-GAL4/UAS-mCD8:GFP fly antennae were used for immunostaining. (B) Coexpression of dTULP in labellar gustatory receptor neurons. Poxn-GAL4/UAS-EGFP fly labella were used for immunostaining. (C) dTULP expression in the femoral chordotonal organ which is marked with F-GAL4/UAS-mCD8:GFP. (TIF) [file pgen.1003814.s002.tif]

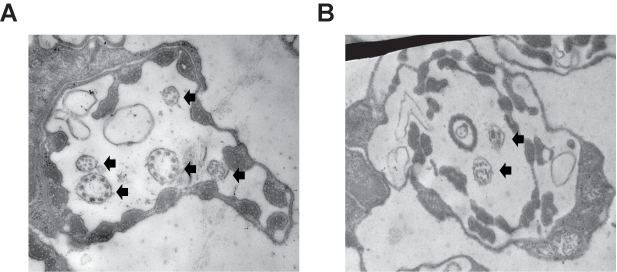

Supplement: Figure S3 — Transmission electron microscopy showing two different types of abnormal scolopidia structure in flies missing dTULP. (A) Abnormal number of chordotonal cilia was observed in 6.2% of examined scolopidia in dTulp1 flies. Arrows indicate ciliary axonemes. (B) The chordotonal cilia were found outside the cap structure in 3.1% of examined scolopidia in dTulp1 flies. Arrows indicate cilia outside the cap. (TIF) [file pgen.1003814.s003.tif]

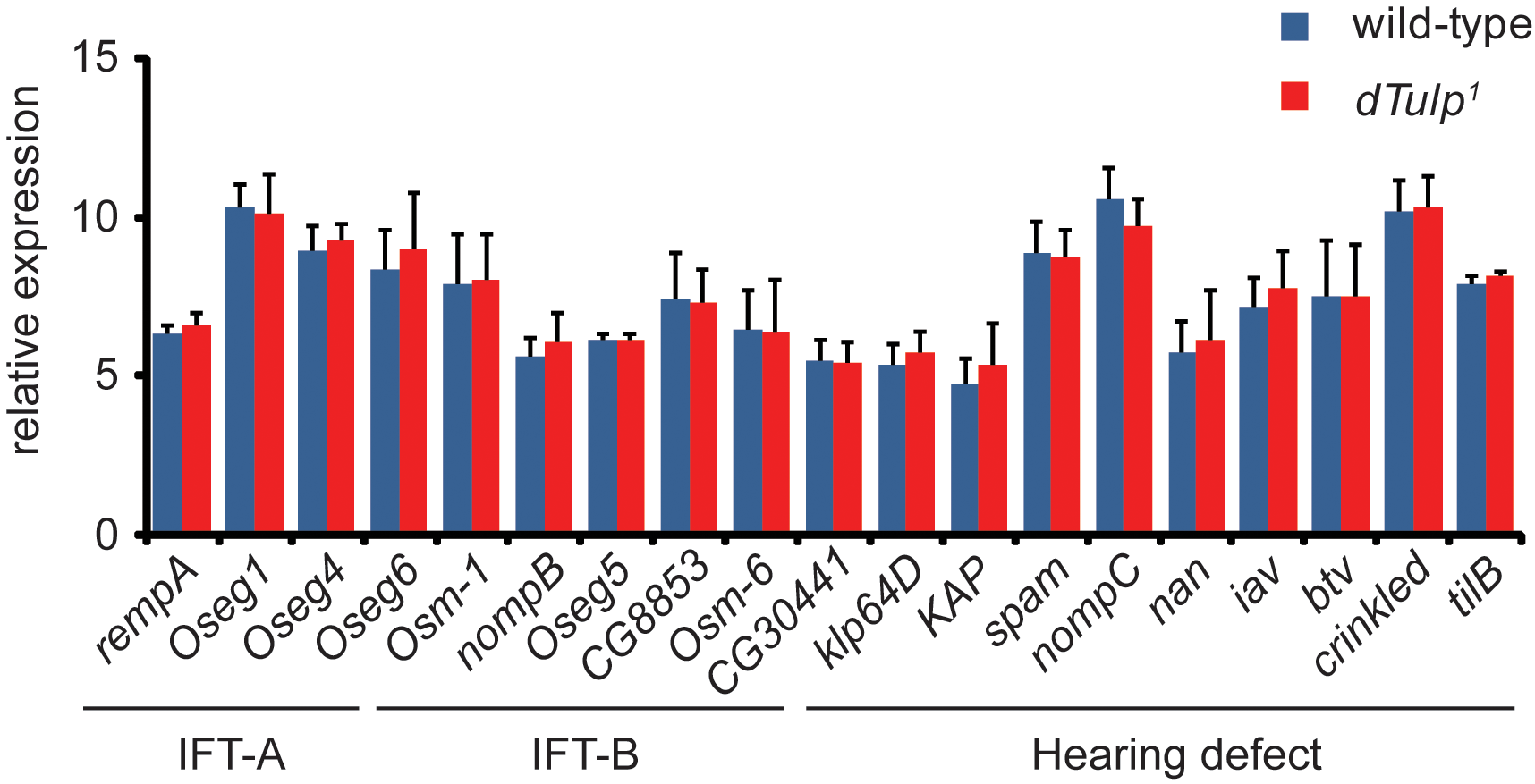

Supplement: Figure S4 — Comparison of mRNA expression of IFTs and hearing-related genes between wild-type and dTulp1 flies. Antennae extracts from wild-type and dTulp1 flies were used for quantitative PCR analyses. Blue and red bars represent wild type and dTulp1, respectively (n = 4–7). Error bars indicate SEM. A two-tailed Student's t test was used to test the statistical significance between wild type and dTulp1. (TIF) [file pgen.1003814.s004.tif]

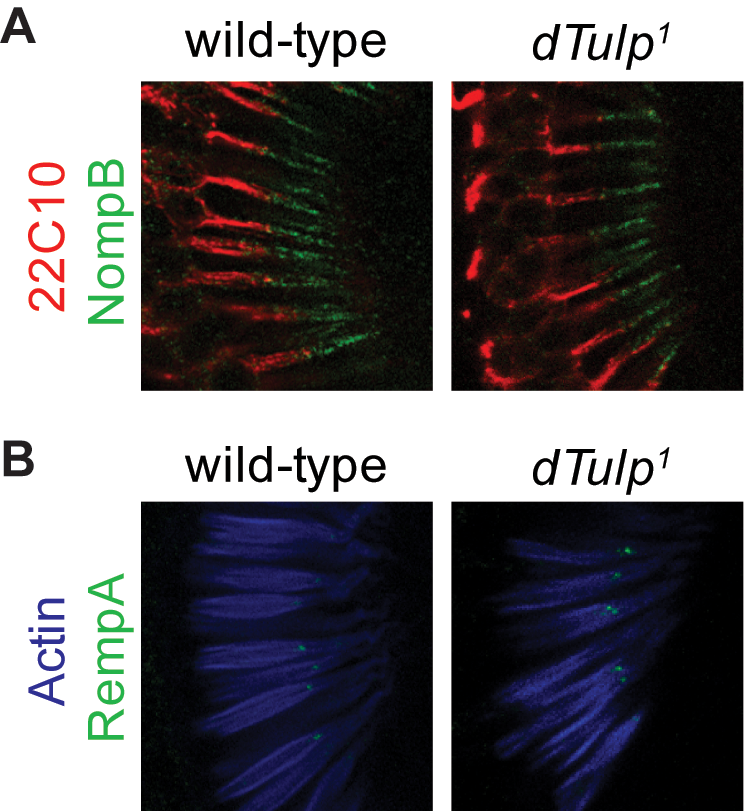

Supplement: Figure S5 — IFT localization in the dTulp1 mutant. (A) Immunostaining of NompB (anti-GFP) counterstained with 22C10 antibodies on antennae of wild type (dTulp1/+;NompB-GFP/+) and the dTulp1 mutant (dTulp1/dTulp1;NompB-GFP/+). (B) Confocal imaging of antennae expressing RempA-YFP from wild-type (dTulp1/+;RempA-YFP/+) and dTulp1(dTulp1/dTulp1;RempA-YFP/+) flies counterstained with phalloidin. (TIF) [file pgen.1003814.s005.tif]

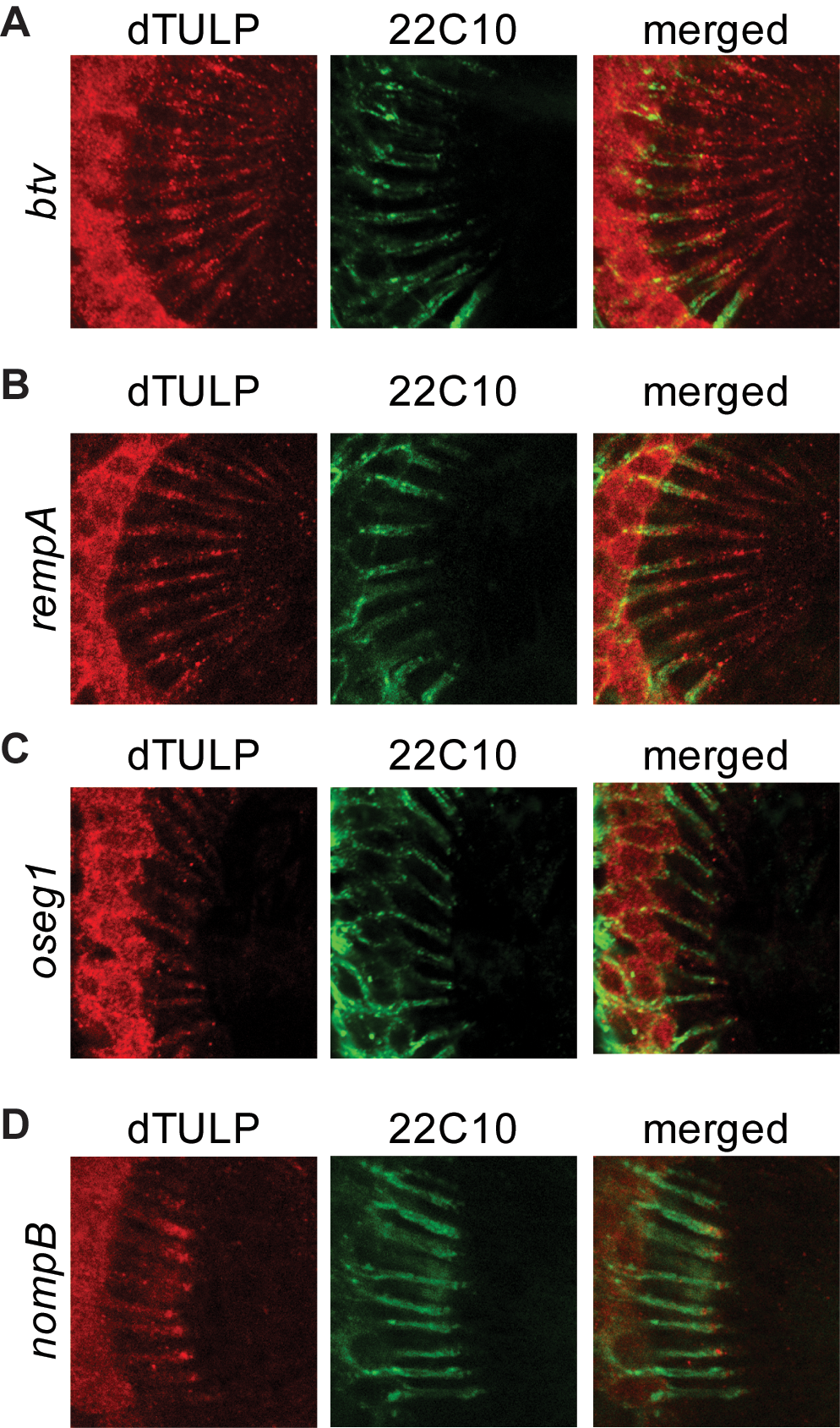

Supplement: Figure S6 — dTULP localization in IFT mutants. (A–D) Immunostaining of dTULP counterstained with 22C10 antibodies on antennae from indicated IFT-related mutants. (A) Ciliary localization of dTULP in beethoven (btv5P1). (B) Ciliary localization of dTULP in rempA (rempA1). (C) Ciliary localization of dTULP in oseg1 (oseg1EP3616). (D) Ciliary localization of dTULP in nompB (nompB1). (TIF) [file pgen.1003814.s006.tif]

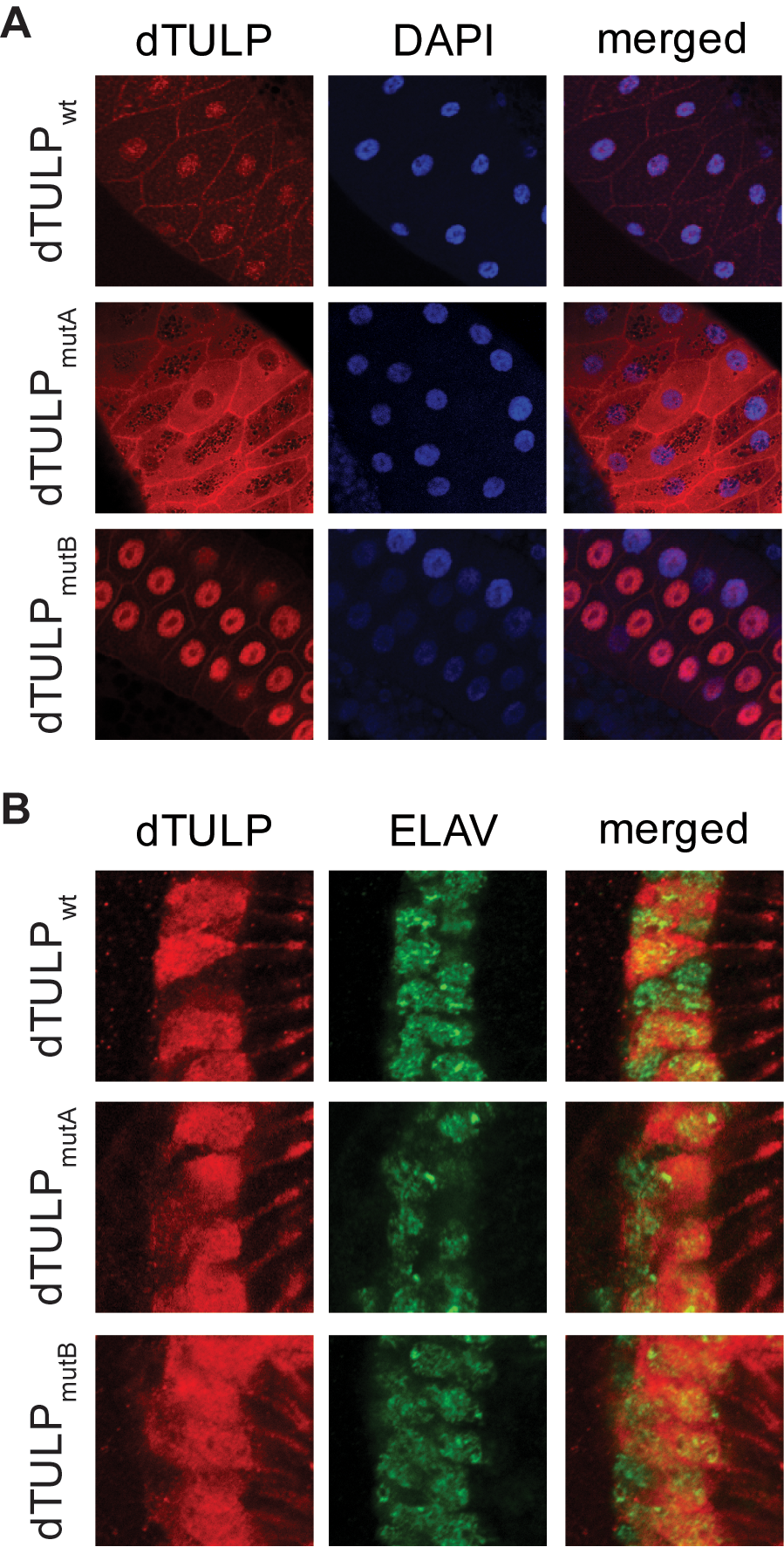

Supplement: Figure S7 — Intracellular localization of different mutant forms of dTULP. (A) Confocal imaging of a third instar larval salivary gland expressing dTULPwt, dTULPmutA, and dTULPmutB. Dissected tissues were immunostained using dTULP antibodies and counterstained with DAPI to visualize the nuclei. AB1-GAL4 was used to drive salivary gland expression of each transgenes. (B) Confocal imaging of the second antennal segment expressing dTULPwt, dTULPmutA, and dTULPmutB. Dissected antennae were immunostained using dTULP and ELAV antibodies which labeled neuron nuclei. (TIF) [file pgen.1003814.s007.tif]

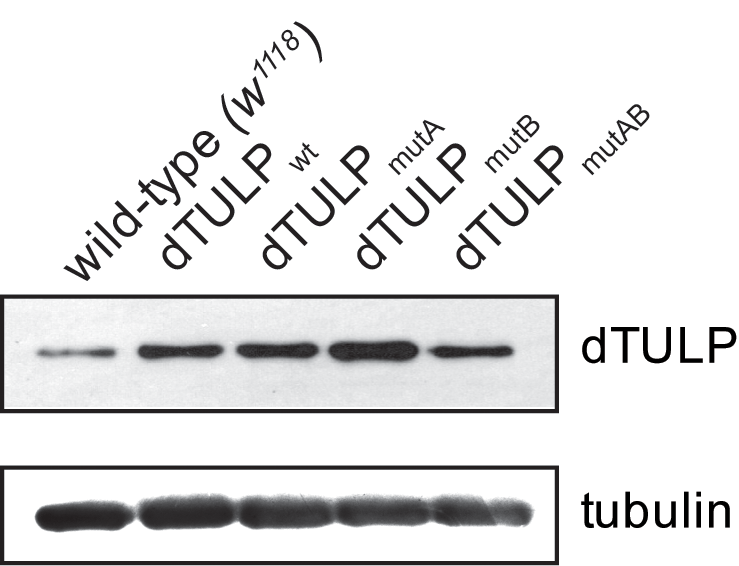

Supplement: Figure S8 — Expression level of various forms of dTULP in the second antennal segment. Western blot probed with antibodies to dTULP and tubulin. Samples were prepared from antennae of the indicated flies. Genotypes are wild-type (w1118), dTulp1,F-GAL4/dTulp1;UAS-dTulpwt/+, dTulp1,F-GAL4/dTulp1;UAS-dTulpmutA/+, dTulp1,F-GAL4/dTulp1;UAS-dTulpmutB/+, dTulp1,F-GAL4/dTulp1;UAS-dTulpmutAB/+. (TIF) [file pgen.1003814.s008.tif]
