# Supplementary material for: dTULP, the Drosophila melanogaster Homolog of Tubby, Regulates Transient Receptor Potential Channel Localization in Cilia
Source: PLoS Genet. 2013 Sep 19;9(9):e1003814. doi: 10.1371/journal.pgen.1003814 (PMC3778012; doi:10.1371/journal.pgen.1003814)
Supplement: Table S1 — Sequence information of real-time PCR primers. (DOCX) [file pgen.1003814.s009.docx]

| **Target** | **Forward (5’→3’)** | **Reverse (5’→3’)** |
| --- | --- | --- |
| *rempA* | TGGTTCTCGCAGGTAAAGATACTCT | CGTAATGCCTCGCCAAGTG |
| *Oseg1* | GTATTCGCACGGTGACTCCATA | TGATCAGCCGACCAAAAGG |
| *Oseg4* | GGCCGGAACGGATGGA | GTCTGGTTCATCGACAAATTGG |
| *Oseg6* | GCATACTGCCCGACGTTAGATAT | CATGATCTGGCACGGTAATAATG |
| *Osm-1* | CCACCGCAGATCGTCATATTCTA | CGGATGACATAGGAGTTCTTACCA |
| *nompB* | ATGATGGGTATAATTGGTGCATTG | TTTGTCGGAGATATACTAAGGCTT |
| *Oseg5* | CGACTCGTGCTACAGCATCTG | GCCAAATTCCGTCGTATCCA |
| *CG8853* | CAGAGCTCAACGAGGTCATGAT | GGCGATCGCTTTCCTGATT |
| *Osm-6* | GGAACAAAATGATGCGGAACTC | TTCAGCACATCGAATTCATCCT |
| *CG30441* | GCATTTATTCAGCGCCAGAAC | TCGCTGTAAAAACTGCAATTCG |
| *klp64D* | CAGGACTCCCTCGGTGGTAA | TGCTATCCGCTGGACTAATGG |
| *KAP* | TCTCATCCACAAGTCCCAGCTT | GCCGGCTGTGAGTGTTGTTA |
| *spam* | GATTTGCCTGCCTCAGCAA | TGGCACTGGATCCCTGTGTA |
| *nompC* | CGTCAAATTGTTGCTAACAAAACGCGG | TGCAGTTCCGGTTTGTCGACTGGAC |
| *nan* | GAGGCCGAGTATATCTCCAATCC | AGCAGGCACAAATGGAGAATAGTT |
| *iav* | AGGCGCCCGAGATGAAA | ATCGATGAGATCACCACCTCTTTT |
| *btv* | GATAAGTGGACGGGAGGGTTTT | CGAAGTCTTAGCTCACCGCTTT |
| *crinkled* | TGCACGAGGCTGGCATCTTGCG | TGGTAGGGATTAACGGCAACAAGTATGG |
| *tilB* | CTACAATCTGAACCAGGCCAAAC | TTAGAGAGGTATCCAGGTGTCTGTACA |
| *rp49* | GACCATCCGCCCAGCATACAG | AATCTCCTTGCGCTTCTTGGAGGAG |
